# An Improved Wake Vortex-Based Inversion Method for Submarine Maneuvering State

**DOI:** 10.1155/2023/5632128

**Published:** 2023-02-11

**Authors:** Dechuan Kong, Zutao Yang, Lei Cai, Haojie Chai

**Affiliations:** ^1^School of Artificial Intelligence, Henan Institute of Science and Technology, Xinxiang 453003, China; ^2^School of Information Engineering, Henan Institute of Science and Technology, Xinxiang 453003, China

## Abstract

As the noise reduction performance of submarines continues to improve, it is difficult to detect and track submarines through acoustic detection techniques. Therefore, nonacoustic submarine detection techniques are becoming more and more important. The submarine movement will leave a wake vortex, and the information of the wake vortex can be used to invert the maneuvering state of the submarine. However, the wake vortex is constantly dissipated in the evolution process, and the strength of the wake vortex is constantly reduced, resulting in the gradual weakening of the characteristics of the wake vortex, which makes the inversion of submarine operating state difficult and less accurate. In order to solve the above problems, this paper proposes an improved wake vortex-based inversion method for submarine maneuvering state. Firstly, a random finite set of submarine wake vortex observation features is established to obtain the feature with the highest correlation degree with submarine maneuvering state in the random finite set. Secondly, the multiscale fusion module and attention mechanism are used to re-encode the weak features of the wake vortex image, and the salient features of the wake vortex image are extracted. Finally, the manipulation state of the wake vortex image is retrieved by the extracted salient features. The experimental results show that the average inversion accuracy of the proposed algorithm is improved by 1.27% in terms of manipulating state inversion of weak feature wake vortex images. The algorithm in this paper can realize the inversion of submarine maneuvering state in the case of weak submarine wake vortex image features and incomplete feature information. It provides the basis for the detection technology based on the submarine wake characteristics.

## 1. Introduction

Due to its great depth, long range, and good concealment, submarines have gradually become the marine military equipment vigorously developed by various countries [1]. With the continuous improvement of underwater target concealment performance and the influence of complex marine environment, the difficulty of underwater target discovery is increased [2]. Therefore, it is urgent to develop new underwater target detection technology to improve the detection ability.

**Figure 1:**
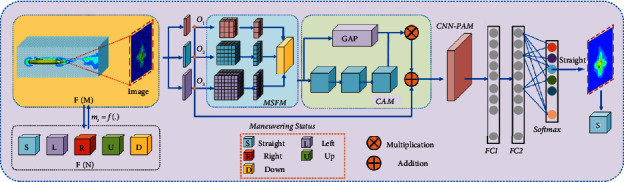
An improved wake vortex-based inversion method for submarine maneuvering state.

Submarines are usually highly covert and difficult to detect. In the process of submarine movement, the interaction between the submarine and the surrounding water medium will form the wake vortex [3, 4]. These wake-vortices can be maintained for a long time in the water, and the steering state of the submarine can be retrieved through the wake -vortices [5–7]. However, the submarine wake vortex is constantly dissipated in the evolution process [8], and the strength of the wake vortex is constantly reduced, resulting in the gradual weakening of the characteristics of the wake vortex, which makes the inversion of the submarine steering state difficult and low accuracy [9]. In order to solve the above problems, this paper proposes an improved wake vortex-based inversion method for submarine maneuvering state. This is shown in Figure 1. This method can be used to retrieve the maneuvering state of submarine when the features of the wake vortex image are weak and the feature information is incomplete.

For this paper, the main contributions are as follows:

1. Construct the submarine wake vortex image observation feature model, and use random finite set to represent the submarine wake vortex observation feature set. This method can effectively solve the problem of stochastic change of wake vortex characteristics caused by uncertainty evolution.
2. The weak feature wake vortex extraction network is established in this paper. The multiscale fusion module and continuous attention module are used to learn the salient features of submarine wake vortex image, which improves the feature extraction accuracy of weak feature wake vortex image.
3. The inversion method of submarine maneuvering state by wake vortex is proposed. The inversion network of submarine maneuvering state is constructed, and the model is trained by Perceptual loss function and Cross-Entropy loss function to achieve accurate judgment of submarine maneuvering state.

## 2. Related Work

Submarines produce vortices in the wake of the submarine during underwater navigation. Based on the characteristics of the submarine wake vortex, it can be detected. Literature [10] demonstrates that the in situ thermohaline distribution of submarine wake contains the key information of submarine detection. Using the method of large eddy simulation, a stratified temperature and salt wake model was established to realize submarine detection according to the change of wake temperature and salinity. In literature [11], submersion test and flow around self-propelled SUBOFF submarine model were conducted. The results show that the submarine interacts with the surrounding water to produce wake flow, and the size, speed, and course of the submarine can be judged according to the characteristics of wake flow. The literature [12] characterizes the submarine wake by means of large eddy simulations and compares it with experimental results of particle image velocimetry, showing that the submarine wake is rich in coherent structure and important features of the submarine parameters. In the literature [13], a numerical simulation method of strongly stratified flow based on a two-layer model was proposed to numerically simulate the near-field motion of the SUBOFF submarine model in the strongly stratified flow, and it was confirmed that the submarine would generate an internal wave wake when sailing underwater, and the effects of the density leap layer, water depth, and airspeed on the internal wave wake were also analyzed. In the literature [14], the vortex structure and wake in homogeneous and stratified fluids were studied, and a thermocline model was proposed to solve the density-varying stratified fluid, and an improved delayed separation vortex simulation method was used to solve the coherent vortex structure and turbulent wake accurately and efficiently, which laid the foundation for realizing the detection of submarines using wake information in a real marine environment. The literature [15] proposes that the interaction between a moving submarine and seawater produces a characteristic wake and demonstrates the feasibility of using visible polarization imaging to detect the wake of a submarine. In the literature [16], the range and intensity of electromagnetic features in the near-field wake of a submarine were obtained by numerical simulation, and it was proposed that the anomalous electromagnetic field in the wake could be used as a scheme to detect submarines. The above studies demonstrate the feasibility of using wake stream for submarine detection from different aspects, and provide a theoretical basis for using wake information for submarine maneuvering state inversion in this paper.

However, the wake can be influenced by evolutionary factors and is uncertain. The literature [17] proposes that the submarine wake evolution can be divided into three periods, and the characteristics of the wake change randomly in different periods. The literature [18] investigated the wake characteristics of a model submarine navigating in a uniform linearly stratified fluid and demonstrated that the stratified fluid affects the formation and evolution of the submarine wake. In the literature [19], the wake vortex evolution was studied by particle image velocimetry and the change process of the wake vortex structure was discussed. In the literature [20], the large-scale coherent vortex structure and its evolution were studied, and it was found that the wake vortex scale grows to a maximum and then undergoes two stages of decay.

The evolution of the submarine wake has led to a gradual weakening of the wake characteristics, which poses difficulties in using wake information to invert the submarine maneuvering state.

Inversion of target states parameters with wake information. In the literature [21], based on the analysis of the joint linear Kelvin wake kinematics and water-wave dispersion relationship, a hybrid method is proposed to decompose and reconstruct the ship wake features in the spectral, spatial, and SAR image domains, which improves the extraction efficiency of the ship wake information and the estimation accuracy of the ship parameters. In the literature [22], the possibility of using wake information for underwater vehicle detection was investigated and an algorithm for identifying the shape, size, speed, and dive depth of underwater vehicles using surface waves was proposed. In the literature [23], a sea surface target motion parameter estimation algorithm was proposed to perform a two-level low-rank plus sparse decomposition of the wake in SAR images using Radon transform, which effectively improves the detection accuracy of wake. In the literature [24], a ship wake detection method for complex marine environments is proposed, where waves and ship wake are superimposed to simulate real sea surface SAR images to improve the adaptability of the wake detection algorithm. In the literature [25], a CNN-based optical image wake detection method is proposed and a novel wake detector (WAKENET) is designed to improve the accuracy of wake detection. In the literature [26], an approximate method for calculating the wake of a ship is proposed, which can quickly predict the ship parameters. In the literature [27], a ship speed estimation method based on the two-dimensional spectrogram of SAR image wake is proposed, which does not require a priori knowledge of SAR parameters and improves the accuracy of the estimation results. In the literature [28], the recognition features of the ship wake in the SAR images of the Yellow Sea were statistically analyzed and the ship motion parameters were extracted, and the results showed that the ship motion parameters could be accurately obtained based on the ship wake.

The existing research addresses the problem of weak target characteristics during target detection. The literature [29] improved the correlation filtering algorithm to effectively solve the problem of target tracking affected by occlusion conditions. The literature [30] designs a multifeature fusion method and establishes the correlation between multichannel features and correlation filters. Experiments show that the algorithm effectively improves the target tracking accuracy. Aiming at the problems of structure disorder and texture detail blur in image restoration, the literature [31] proposes an image restoration network driven by multilevel attention mechanism. By compressing the advanced features of the full resolution image, finegrained image restoration, and reconstruction can be achieved. The literature [32] adapts to a more complex traffic sign detection environment by adding more realistic traffic scene images. Experiments show that the algorithm has higher robustness and real-time performance. The literature [33] proposes an underwater distortion target recognition network (UDTRNET). Underwater weak feature targets are recognized more accurately by fusing salient features and spatial semantic features. A salient target detection network (TSEID) is proposed in the literature [34]. Using a dual-stream encoder and an interactive decoder to balance the feature domain differences, the algorithm effectively improves the salient features of targets and enhances the detection performance of weak feature targets. A hierarchical feedback network containing multilevel spatial pyramids is proposed in the literature [35]. Context-aware multiscale features with different receptive field sizes are obtained, and the multiscale information is decoded using an attention mechanism. The algorithm has strong robustness for weak target recognition in different scenarios. An attention-intensive spatial pyramid module was designed in the literature [36]. Dilated convolution is used to acquire local and global features, thereby improving its performance for detecting weak feature targets. The literature [37] proposes a dense multiscale inference network (DMINET). Through the convolution operation of different receptive field and dense connection, the multiscale context features are effectively captured and utilized, which improves the ability of target detection in complex background. For incomplete target representation data, a GAN-meta-learning-based target recognition method is proposed in the literature [38] to make up for the missing target information. A good generalization capability is demonstrated by experiments. A pixel-by-pixel contextual attention network (PICANET) is proposed in the literature [39]. The attention graph is generated in the contextual region of each pixel, and the saliency features of the target are improved by selectively combining features of useful contextual locations to construct attentional contextual features. Experiments show that the algorithm has excellent generalization ability. In the literature [40], a dual attention residual module and hierarchical feature screening module are designed to achieve residual refinement and obtain more global contextual knowledge. The algorithm effectively improves the detection performance of weak feature targets.

## 3. Proposed Method

### 3.1. Wake Vortex Observation Model

**Figure 2:**
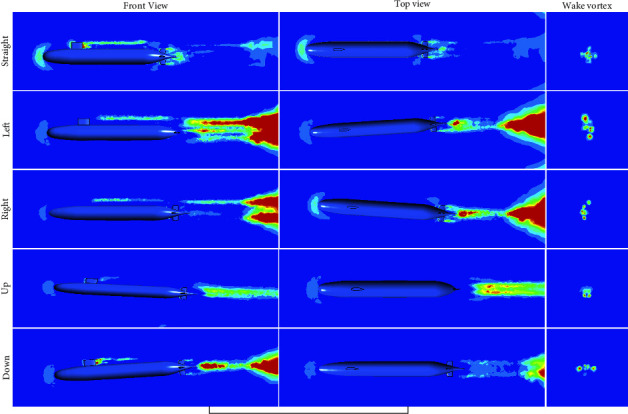
Evolution of SUBOFF wake vortex under different maneuvering conditions.

The submarine has straight, yaw, pitch, and other maneuvering movements underwater, and the submarine would produce wake vortex in the wake area when sailing underwater. Under different operating states, the shape characteristics of the submarine wake vortex are different [41]. In this paper, the wake evolution process of the fully attached SUBOFF submarine model under different maneuvering states is simulated, as shown in Figure 2.

In the inversion of submarine maneuvering state, the evolution of the submarine wake vortex is uncertain, leading to random changes in the characteristics of the wake vortex [42]. The observed submarine wake vortex is modeled as set *M*, and the submarine steering state is modeled as set *N*. The observation feature set of submarine wake vortex and the manipulation state set of submarine are, respectively, expressed as follows:

(1) $$\begin{array}{c} M=\left\{m_{1},m_{2},\ldots ,m_{k}\right\}\in F\left(M\right)\text{,}\\ N=\left\{n_{1},n_{2},\ldots ,n_{k}\right\}\in F\left(N\right)\text{,} \end{array}$$

where *m*_*k*_ is the *k* th class of wake vortex features; *F*(*M*) denotes the observation space of wake vortex features; *n*_*k*_ is the *k* th class of submarine maneuvering state; and *F*(*N*) denotes the submarine maneuvering state space. The observation space of wake vortex features and submarine maneuvering state space contain all submarine wake vortex features information and all submarine motion state forms, respectively.

In the submarine observation feature set *M*, the set element *m*_*i*_(*i*=1,2,3,…, *k*) varies randomly, while the set base |*M*|=*k* also varies randomly. Submarine observation feature set *M*={*m*_*i*_}_*i*=1_^|*M*|^ is regarded as random finite set, and the probability density is

(2) $$\begin{array}{c} \begin{array}{c} p\left(M\right)=p\left(\left|M\right|\right)\left(\left|M\right|\right)!U^{\left|M\right|}p_{\left|M\right|}\left(m_{1},\ldots ,m_{\left|M\right|}\right) \end{array}\text{,} \end{array}$$

where *p*(|*M*|=*k*)=*p*(*k*) is the discrete basis distribution, *U* denotes the unit hyperspace, and *p*_*k*_(*m*_1_,…, *m*_*k*_) is the symmetric joint eigen density for a given basis |*M*|=*k*.

The probability that the feature set *M* can characterize the maneuvering state of the submarine is obtained, and the probability density associated with the shape features of the submarine wake vortex in the observed feature set *M* is calculated. The features corresponding to the maximum correlation probability density are filtered and fed into the submarine wake vortex feature extraction network. It can be expressed as follows:

(3) $$\begin{array}{c} m_{t}=f\left(p_{\max}\left(M\right)\right)\text{,} \end{array}$$

where *m*_*t*_ denotes the feature with the highest probability associated with the shape feature in the submarine observation feature set *M*; *f*(∙) denotes the mapping function of the probability density of the submarine observation feature set *M* to the set elements.

### 3.2. Weak Feature Wake Vortex Extraction

According to the wake vortex description model, it is known that the wake vortex uncertainty evolution would cause the wake vortex features to become weaker. Therefore, the encoder network is used to re-encode the features of the wake vortex image, reduce the interference of the uncertain evolution factors on the wake vortex features, and realize the efficient extraction of the weak features of the wake vortex.

**Figure 3:**
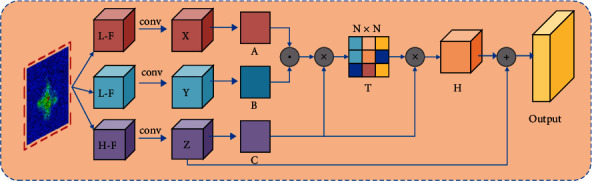
Multiscale feature fusion module (MSFM).

The receptive field of the input wake vortex image is learned by three branches, and its convolution kernel size is 3 × 3, 5 × 5, and 7 × 7, respectively. In order to measure the semantic correlation between the output feature maps under different convolution kernel sizes, reduce the semantic information interference between the feature maps, and reduce the semantic gap, a multiscale feature fusion module (MSFM) is designed. The multiscale feature fusion module is shown in Figure 3.

In the multiscale feature fusion module, the correlation between pixels in different feature maps is calculated by matrix multiplication in order to reduce the computational burden. And the correlation is used as the weight vector of the feature map output by larger convolution kernel. As shown in the following equation,

(4) $$\begin{array}{c} \begin{array}{c} t_{\alpha \beta }^{\gamma }=\frac{\exp\;\left(A_{\alpha }\cdot B_{\beta }\cdot C_{\gamma }\right)}{\overset{N}{\underset{\alpha ,\beta =1}{\sum }}\exp\;\left(A_{\alpha }\cdot B_{\beta }\cdot C_{\gamma }\right)} \end{array}\text{,} \end{array}$$

where *t*_*αβ*_^*γ*^ is the correlation between the *α* th position and the *β* th position in the output feature map of the smaller convolution kernel and the *γ* th position in the larger convolution kernel output feature map. *N* pixels are present in the image. *A* and *B* denote the feature maps obtained by convolving and reshaping the output of the smaller convolution kernel. *C* denotes the feature maps obtained by convolving and reshaping the output of the larger convolution kernel. The multiscale fusion calculation process is show in the following equation:

(5) $$\begin{array}{c} G_{\gamma }=\xi \overset{N}{\underset{\alpha ,\beta =1}{\sum }}\left(t_{\alpha \beta }^{\gamma }c_{\alpha \beta }\right)+Z_{\gamma }\text{,} \end{array}$$

where *ξ* denotes the learning weight; c_*αβ*_ denotes the *α* th position and the *β* th position of the lower feature map; and *Z*_*γ*_ denotes the *γ* th position of the upper feature map.

**Figure 4:**
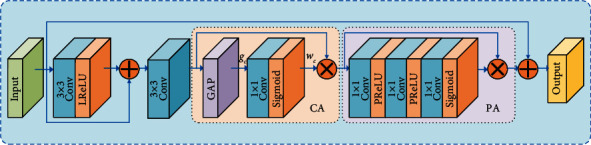
Abstract feature extraction process.

To avoid extracting more irrelevant features and to better adapt to the complex and changing underwater environment, the attention mechanism is used to improve the feature extraction accuracy of the model. The feature attention module is shown in Figure 4.

Feature attention modules use local residual structures to pass output features into the convolution layer. Then the output results and the input features are superimposed by converting the Leaky ReLU activation function to nonlinearity. The superimposed features are passed through the convolutional layer and used as the input to the channel attention. In channel attention, the fast one-dimensional convolution with kernel size *d* is used as the fully connected layer, and the learning parameters are shared between channels. As shown in the following equation,

(6) $$\begin{array}{c} \begin{array}{c} W_{c}=\sigma \left(Conv1D_{d}\left(g_{c}\right)\right)\text{,} \end{array} \end{array}$$

where *W*_*c*_ denotes the channel weights, *σ* denotes the sigmoid function, Conv1*D* denotes the one-dimensional convolution, and *g*_*c*_ denotes the result of global average pooling of the channel attention input features.

The channel attention input features are multiplied pixel by pixel with the channel weights and then passed to the pixel attention layer. The pixel attention layer consists of the PReLU activation function and the Sigmoid activation function. The pixel weights are obtained through the pixel attention layer, and finally the obtained pixel weights are used to weigh the pixel attention input features.

The continuous attention module (CAM) is used to further improve the performance of the wake vortex feature coding network. However, continuous attention module will increase network depth, and excessive increase of network depth will lead to weakened expressive ability [43]. The salient features are learned adaptively and the shallow feature information is transferred to the deep layer. Global average pooling is used to adaptively obtain the feature vectors of different channels, and then the feature vectors are multiplied with the output features of the continuous attention module. As shown in the following equation,

(7) $$\begin{array}{c} \begin{array}{c} F_{M}=S_{G}\left(O_{c}\right)⊗S_{CAM}\left(O_{c}\right)⊕O_{s}\text{,} \end{array} \end{array}$$

where *F*_*M*_ is the output of adaptive learning, *S*_*G*_ and *S*_CAM_ perform global average pooling and continuous attention operations, respectively, *O*_*c*_ is the input feature of the continuous attention module, and *O*_*s*_ denotes shallow information.

### 3.3. State Inversion Network Model

In this paper, CNN is used as the basic network structure, and the wake vortex feature coding network is embedded in the backbone network model, so as to establish the submarine maneuvering state inversion network. Specifically, the submarine maneuvering state retrieval network in this paper can be divided into two stages. In the first stage, aiming at the problem that the characteristics of the wake vortex are weakened and the feature information is incomplete caused by the uncertain evolution factors of the wake vortex, the encoder network is used to re-encode the features of the wake vortex image to achieve the efficient extraction of the weak features of the wake vortex. In the second stage, the feature map obtained from the wake vortex feature coding network is used as input, and the submarine steering state is retrieved in the fully connected layer.

**Figure 5:**
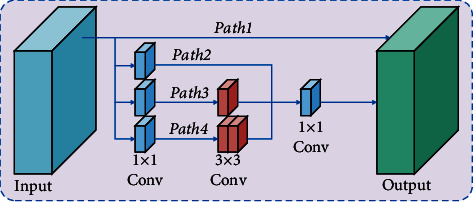
CNN path adjustment module (CNN-PAM).

In addition, to avoid overfitting and gradient disappearance during training, a CNN path adjustment module (CNN-PAM) is added to the submarine maneuvering state retrieval network, as shown in Figure 5.

The output results of the wake vortex feature coding network are transferred to the module, so as to reduce the number of parameters of the submarine maneuvering state inversion network, reduce the training time of the network, and improve the accuracy of the submarine maneuvering state inversion. Finally, the output of the CNN path adjustment module is taken as input through two fully connected layers (FC1 with 1024 output neurons and FC2 with 8 output neurons), and the Softmax function is used to predict the label of the submarine manipulation state.

### 3.4. Loss Function

The CFD technique is used to simulate different maneuvering states of the submarine during underwater motion and to obtain information on the submarine wake vortex data. The wake vortex images at different positions of the submarine wake are intercepted and formed into a training dataset *T*_data_={(*f*_1_, *l*_1_), (*f*_2_, *l*_2_), ⋯, (*f*_*i*_, *l*_*i*_)}, *i* ∈ *R*, where *f* denotes the acquisition of the submarine wake vortex image information and *l* denotes the submarine wake vortex image label. The submarine maneuvering state inversion network is trained by the training dataset *T*_da ta_, and the optimal weight parameters of the inversion network are calculated to ensure that the model has a good generalization capability.

Perceptual loss and cross-entropy loss are used as loss functions. The weak features of the wake vortex image are extracted using the perceptual loss function. The perceptual loss function can be expressed as follows:

(8) $$\begin{array}{c} L_{s}=\frac{1}{CWH}\overset{C}{\underset{c=1}{\sum }}\overset{W}{\underset{w=1}{\sum }}\overset{H}{\underset{h=1}{\sum }}{\left(P\left(O_{c,w,h}^{c}-P\left(O_{c,w,h}^{g}\right)\right)\right)}^{2}, \end{array}$$

where *C*, *W*, and *H* denote the channel, width, and height of the image, respectively. *O*_*g*_ denotes the wake vortex significant feature map output by the wake vortex weak feature extraction network. *P* denotes the nonlinear transformation.

The cross-entropy loss function is used to portray the distance between the actual output of the inverse network submarine manipulation state and the desired output. Probabilities are indicated more closely when cross-entropy is smaller. Suppose, the actual output of a sample is *p* and the expected output is *q*, then there is a deviation between *p* and *q*. By continuously training the network model iteratively, making *p* closer and closer to *q*. The mathematical expression is as follows:

(9) $$\begin{array}{c} H\left(p,q\right)=-\sum_{x}p\left(x\right)\log\;q\left(x\right)\text{.} \end{array}$$

Therefore, the total loss function is as follows:

(10) $$\begin{array}{c} L_{loss}=L_{s}+\lambda H\left(p,q\right)\text{,} \end{array}$$

where *λ* is a weighting factor that regulates the ratio of the two loss functions.

## 4. Experimental Results and Analysis

### 4.1. Experimental Dataset

In this paper, CFD technology was used to simulate the wake evolution characteristics of the fully attached SUBOFF model under different manipulation states, and Submarine Wake Dataset (SWD) used in this experiment was constructed. The SWD dataset has 6797 label images. The images in the dataset contain five control states during the underwater movement of the submarine, namely, straight, left, right, up, and down. The SWD dataset was randomly divided into training dataset and test dataset according to the ratio of 7 : 3. The training dataset included 968 straight images, 939 left-skewed images, 950 right-skewed images, 954 uptilted images, and 949 downtilted images. The test set consists of 414 straight images, 402 left-skewed images, 407 right-skewed images, 408 uptilted images, and 406 downtilted images. The SWD dataset statistics are shown in Table 1.

### 4.2. Experimental Platform and Parameters

The training and testing of this experiment were carried out on PyTorch platform under Ubuntu18.04 operating system. The device configuration is as follows: 64 GB RAM and two RTX 2080Ti GPUs. The experimental code is mainly based on Python language, including data preprocessing and algorithm implementation.

Stochastic gradient descent (SGD) optimizer is used for model training. The initial learning rate is 0.001. The momentum parameter is 0.9. The weight decay is 0.0005. The total number of epochs for training is 300.

### 4.3. Experimental Results

This paper presents an improved wake vortex-based inversion method for submarine maneuvering state. Due to the uncertainty of evolution, facing the problem of weakening the features of submarine wake vortex image caused by evolution factors, Gaussian noise and random pixel zeroing are, respectively, added to the wake vortex image to characterize the weak feature of submarine wake vortex image. Therefore, this paper sets up three groups of submarine maneuvering state inversion simulation experiments. The effectiveness of the proposed method is verified by comparing the results of submarine maneuverability state inversion with MFENet [44], SA-SPPN [45], DAFNet [46], and APAN [47] algorithms. The evaluation criteria of the algorithm are mean average accuracy (mAP) and overall accuracy (Acc).

#### 4.3.1. Significant Wake Vortex Image Inversion Results

**Figure 6:**
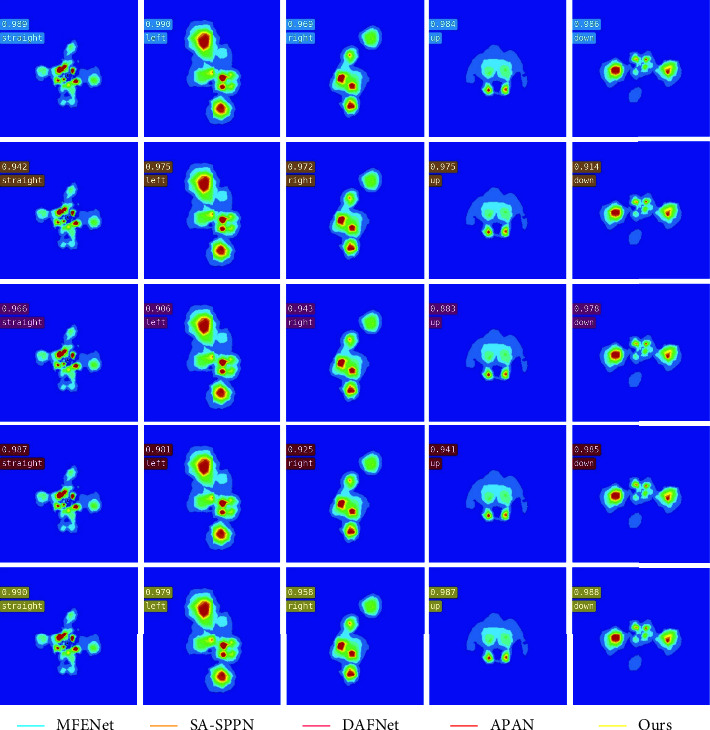
Inversion results of significant wake vortex image manipulation state.

Figure 6 shows the inversion result of manipulation state of submarine salient wake vortex image by the proposed algorithm. In Figure 6, each row of images represents the inversion accuracy of each algorithm for different control states of the submarine, which are five types of control states: straight, left, right, up, and down.  Table 2 shows the average accuracy and overall accuracy of the algorithm for retrieving the manipulation state of submarine salient wake vortex image. It can be seen from the inversion results of the five algorithms on submarine maneuvering state that the average accuracy of the proposed algorithm is the best, which is 0.7857. The algorithm in this paper is also the highest in the inversion accuracy of the maneuvering state of submarine left and right deviation, which were 0.7910 and 0.7173, respectively. However, the overall accuracy of the proposed algorithm is slightly lower than that of MFENet, with an overall accuracy of 0.7785. The MFENet algorithm has the highest inversion accuracy of 0.7730 for the maneuvering state of the submarine up. The SA-SPPN algorithm has the highest inversion accuracy of 0.9102 for the maneuvering state of the submarine straight. The DAFNet algorithm is slightly higher than the algorithm in this paper in terms of inversion accuracy of the maneuvering state of the submarine down, which is 0.8230. In terms of overall accuracy, the MFENet algorithm has the best effect, which is 0.7876. From the above analysis, it is shown that the proposed algorithm is the best in the average accuracy of submarine maneuvering state inversion, but it is not ideal in the inversion of submarine direct navigation and upturn maneuvering state.

#### 4.3.2. Weak Feature Wake Vortex Image Inversion Results

**Figure 7:**
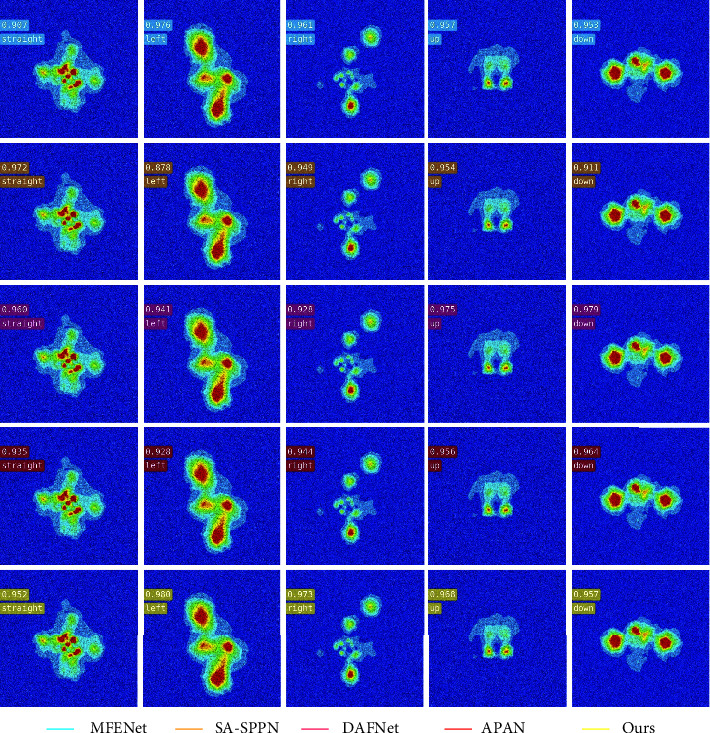
Result of inversion of manipulated state of Gaussian noise wake vortex image.

Figure 7 is the inversion result of manipulation state of submarine wake vortex image with Gaussian noise by the proposed algorithm. The five columns in the figure, respectively, represent the inversion accuracy of the five control state types of the submarine under different algorithms: straight, left, right, up, and down.  Table 3 shows the average accuracy and overall accuracy of manipulating state inversion of submarine wake vortex image with Gaussian noise. It can be analyzed from the table that the manipulation state inversion result of the algorithm in this paper is the best when facing the submarine wake vortex image with weak features. The average accuracy and overall accuracy of submarine maneuvering state inversion are 0.7645 and 0.7572, respectively. The average inversion accuracy of the proposed algorithm is improved by 1.27%. In addition, the algorithm in this paper still maintains the highest inversion accuracy for the maneuvering state of the submarine left deviation and right deviation, which are 0.7501 and 0.6969, respectively. However, SA-SPPN algorithm has the highest inversion accuracy of 0.8789 for direct navigation of submarine. The DAFNet algorithm is higher than the algorithm in this paper in terms of maneuver state inversion accuracy for submarine down, which is 0.8095. The APAN algorithm has a slightly higher inversion accuracy of 0.7539 than the proposed algorithm for the inversion of the maneuvering state on the submarine up. From the above data, it can be seen that the proposed algorithm has the highest average accuracy and overall accuracy when retrieving the manipulation state of submarine weak feature wake vortex image.

**Figure 8:**
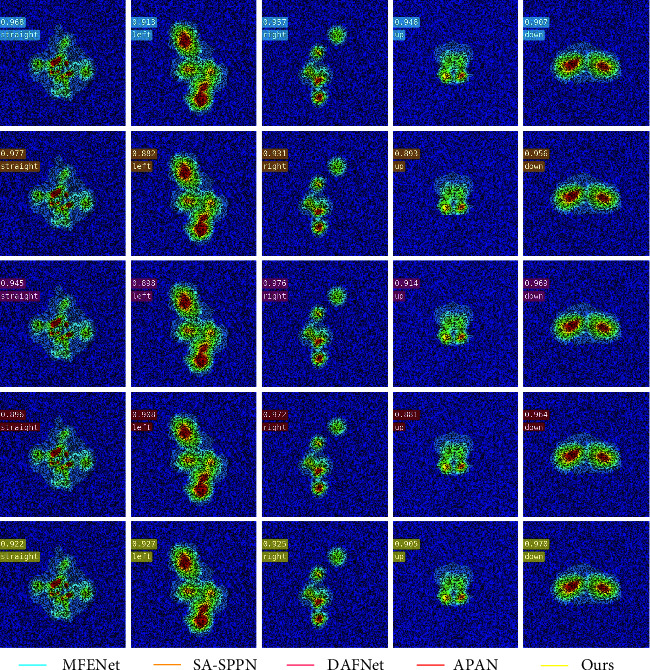
The result of manipulation state inversion of random pixel zeroing wake vortex image.

Figure 8 is the inversion result of manipulation state of submarine wake vortex image with random pixel zeroing by the proposed algorithm. Each column in the figure represents the inversion accuracy of different algorithms for five types of submarine control states: straight, left, right, up, and down.  Table 4 shows the mAP and Acc of manipulating state inversion of submarine wake vortex image with random pixel zeroing. According to the inversion results in Table 4, it can be seen that the MFENet algorithm has the highest mAP of 0.7437 for the inversion of the maneuvering state of the weakly featured wake vortex image of the submarine. The Acc was 0.7403. The DAFNet algorithm has the best Acc of 0.7445. Although the average inversion accuracy of the proposed algorithm is 1.85% lower than that of the MFENet algorithm, the inversion results of the maneuvering state of the submarine with left and down are much better in this paper.

## 5. Conclusion

Due to the influence of evolution factors, the feature information of submarine wake vortex is not complete. It is challenging to invert the maneuvering state of submarine using the weak feature wake vortex image. In this paper, an inversion method for maneuvering state of submarine based on wake vortex is proposed. Firstly, the observation features of the submarine wake vortex are modeled as a random finite set, and the best features of the set are screened out. Secondly, the multiscale fusion module and attention mechanism are used to re-encode the weak features of the wake vortex image, extract the salient features of the wake vortex image, and reduce the interference of uncertain evolution factors on the characteristics of submarine wake vortex. Finally, the manipulation state of the wake vortex image is inverted by the salient features extracted from the wake vortex feature coding network. The experimental results show that the average accuracy of the proposed algorithm is improved by 1.27% when the wake vortex image features are weak. However, the interference of ocean turbulence on submarine wake vortex is not considered in this study, and the correction of submarine wake vortex distortion under the interference of ocean turbulence can be studied in future work.

## Figures and Tables

**Table 1:** SWD dataset statistics.

Class	Training	Testing
Straight	968	414
Left	939	402
Right	950	407
Up	954	408
Down	949	406

**Table 2:** Significant wake vortex image manipulation state inversion mAP and Acc.

Method	Straight	Left	Right	Up	Down	mAP	Acc
MFENet	0.8594	0.7792	0.6929	**0.7730**	0.7541	0.7717	**0.7876**
SA-SPPN	**0.9102**	0.7014	0.7058	0.7051	0.7775	0.7600	0.7648
DAFNet	0.8740	0.7061	0.6672	0.7431	**0.8230**	0.7627	0.7724
APAN	0.7967	0.7735	0.6906	0.7718	0.7939	0.7653	0.7631
Ours	0.8659	**0.7910**	**0.7173**	0.7503	0.8042	**0.7857**	0.7785

Ours are the results of the inversion of the algorithms in this paper. The bold values represent the excellent metrics for each algorithm.

**Table 3:** Gaussian noise wake vortex image manipulation state inversion mAP and Acc.

Method	Straight	Left	Right	Up	Down	mAP	Acc
MFENet	0.8682	0.6626	0.6498	0.7449	0.7081	0.7267	0.7292
SA-SPPN	**0.8789**	0.6503	0.6516	0.6175	0.7421	0.7081	0.7045
DAFNet	0.8723	0.6748	0.6644	0.7380	**0.8095**	0.7518	0.7495
APAN	0.7812	0.6323	0.6469	**0.7539**	0.7352	0.7099	0.7074
Ours	0.8423	**0.7501**	**0.6969**	0.7426	0.7908	**0.7645**	**0.7572**

Ours are the results of the inversion of the algorithms in this paper. The bold values represent the excellent metrics for each algorithm.

**Table 4:** The mAP and Acc of manipulation state inversion of random pixel zeroing wake vortex image.

Method	Straight	Left	Right	Up	Down	mAP	Acc
MFENet	0.8543	0.7154	0.6821	**0.7713**	0.6956	**0.7437**	0.7403
SA-SPPN	**0.8747**	0.6172	0.6705	0.6403	0.7208	0.7047	0.7012
DAFNet	0.8331	0.6486	**0.7214**	0.7023	0.7725	0.7356	**0.7445**
APAN	0.7504	0.6892	0.6869	0.6515	0.7445	0.7045	0.7088
Ours	0.7991	**0.7269**	0.6340	0.6908	**0.7754**	0.7252	0.7146

Ours are the results of the inversion of the algorithms in this paper. The bold values represent the excellent metrics for each algorithm.

## Data Availability

The data used to support the findings of this study are available from the corresponding author upon request.
